# Supplementary material for: Functional analysis in a model sea anemone reveals phylogenetic complexity and a role in cnidocyte discharge of DEG/ENaC ion channels
Source: Commun Biol. 2023 Jan 6;6:17. doi: 10.1038/s42003-022-04399-1 (PMC9822975; doi:10.1038/s42003-022-04399-1)
Supplement: Supplementary file 2 — Supplementary Information [file 42003_2022_4399_MOESM2_ESM.pdf]

# Supplementary Information for

## Functional analysis in a model sea anemone reveals phylogenetic complexity and a role in cnidocyte discharge of DEG/ENaC ion channels

Jose Maria Aguilar-Camacho[1†‡], Katharina Foreman[2†], Adrian Jaimes-Becerra[1], Reuven Aharoni[1], Stefan Gründer[2*] & Yehu Moran[1*]

*Corresponding authors: Stefan Gründer. Email: sgruender@ukaachen.de. Yehu Moran. Email: yehu.moran@mail.huji.ac.il.

**This PDF file includes:**

Supplementary Tables 1-6
Supplementary Figures 1-14
Supplementary References

## SUPPLEMENTARY TABLES

**Supplementary Table 1. Stimuli tested on NeNaCs and DEG-NeNaCs**. NeNaCs are listed in numerical order from left to right. NeNaCs from clade A are shown on a sky blue background, and NeNaCs from clade B on a reddish purple background. Screening stimuli included acidic pH (mostly 6.0 – 5.0), 100 µM amiloride, 10 µM diminazene and reduction of $[Ca^{2+}]_e$ to 10 µM. A "+" sign indicates that current was elicited or inhibited. A "-" sign indicates that no current was elicited or inhibited. NT = Not Tested. For NeNaC23, we did not generate a DEG mutant.

| NeNaC WT | | | | | | | | | | | | | | | |
|---|---|---|---|---|---|---|---|---|---|---|---|---|---|---|---|
| **Tested Stimuli** | **1** | **2** | **3** | **5** | **6** | **7** | **8** | **9** | **10** | **11** | **12** | **14** | **15** | **23** | **24** |
| pH | - | + | - | - | - | - | - | - | - | - | - | + | - | - | - |
| amiloride | - | - | NT | NT | NT | - | + | NT | NT | NT | NT | - | NT | - | NT |
| diminazene | - | - | NT | NT | - | - | + | - | NT | NT | NT | - | NT | - | NT |
| low Ca2+ | - | - | - | - | - | - | + | - | - | - | - | - | - | - | - |
| **DEG NeNaCs** | | | | | | | | | | | | | | | |
| **Tested Stimuli** | **1 G495T** | **2 G516T** | **3 S464T** | **5 G418T** | **6 S423T** | **7 G404T** | **8 G423T** | **9 S425T** | **10 S419T** | **11 A438T** | **12 S430T** | **14 G471T** | **15 G460T** | | **24 G457T** |
| pH | + | + | + | - | - | - | + | - | - | - | - | + | + | | - |
| amiloride | + | + | - | - | - | - | + | - | - | - | - | + | + | | - |
| diminazene | + | + | - | - | - | - | + | - | - | - | - | + | + | | - |

**Supplementary Table 2. Peptides tested on NeNaCs.** Peptide sequences are listed on the left. Superscript numbers denote references. pQ = pyroglutamate. NeNaCs are listed numerically. NeNaC1 and 24 were also co-injected (right column). NeNaCs from clade A are shown on a sky blue background, and NeNaCs from clade B on a reddish purple background. A "-" sign indicates that no current was elicited or inhibited. NT = Not Tested.

| Tested Peptides | 1 | 2 | 3 | 5 | 6 | 7 | 8 | 9 | 10 | 11 | 12 | 14 | 15 | 23 | 24 | 1&24 |
|---|---|---|---|---|---|---|---|---|---|---|---|---|---|---|---|---|
| pQGRFa[1] | - | - | - | - | - | NT | - | - | - | - | - | - | NT | NT | - | - |
| VVLRRYa[1] | - | - | - | | - | NT | - | - | - | - | - | - | NT | NT | - | - |
| WSCSLRPa[1] | - | - | - | - | - | NT | - | - | - | - | - | - | NT | NT | - | - |
| WSCCLRPa[1] | - | - | - | - | - | NT | - | - | - | - | - | - | NT | NT | - | - |
| LVGRWa[1] | - | - | - | - | - | NT | - | - | - | - | - | - | NT | NT | - | - |
| DRTa[1,2] | - | - | - | - | - | NT | - | - | - | - | - | - | NT | NT | - | - |
| pQAGAPGLWa[2] | - | - | - | - | - | NT | - | - | - | - | - | - | NT | NT | - | - |
| pQAGPPGLWa[2] | - | - | - | - | - | NT | - | - | - | - | - | - | NT | NT | - | - |
| 3-L-phenyllactylLRNa[2] | - | - | NT | - | NT | NT | - | NT | NT | NT | NT | - | NT | NT | NT | - |
| 3-L-phenyllactylYRVa[2] | - | - | NT | - | NT | NT | - | NT | NT | NT | NT | - | NT | NT | NT | - |
| pQGLRWa[2] | - | - | NT | - | NT | NT | - | NT | NT | NT | NT | - | NT | NT | NT | - |
| GPRGa[2] | - | - | NT | - | NT | NT | - | NT | NT | NT | NT | - | NT | NT | NT | - |
| pQGRFa[2] | - | - | NT | - | NT | NT | - | NT | NT | NT | NT | - | NT | NT | NT | - |
| pQLFRPa[2] | - | - | NT | - | NT | NT | - | NT | NT | NT | NT | - | NT | NT | NT | - |
| pQLLFRPa[2] | - | - | - | - | - | - | - | - | - | - | - | - | NT | NT | - | - |
| NPPIDLGPAYFHIRa[3] | - | - | - | - | - | - | - | - | - | - | - | - | NT | NT | - | - |
| pQPPIDLSPAAYFHIRa[3] | - | - | - | - | - | - | - | - | - | - | - | - | NT | NT | - | - |
| GPRGGRATEFGPRGa[3] | - | - | - | - | - | - | - | - | - | - | - | - | NT | NT | - | - |
| IPPQGLRFSQWa[3] | - | - | - | - | - | - | - | - | - | - | - | - | NT | NT | - | - |
| MPEQDANPQTRFDa[3] | - | - | - | - | - | - | - | - | - | - | - | - | NT | NT | - | - |
| pQGRFGREDQGRFa[3] | - | - | - | - | - | - | - | - | - | - | - | - | NT | NT | - | - |
| FPPGFHRPa[3] | - | - | - | - | - | - | - | - | - | - | - | - | NT | NT | - | - |
| GPPMIKIPVRHa[3] | - | - | - | - | - | - | - | - | - | - | - | - | NT | NT | - | - |
| pQGRFa[2] | - | - | - | - | - | - | - | - | - | - | - | - | NT | NT | - | - |
| pQAGAPGLWa[2] | - | - | - | - | - | - | - | - | - | - | - | - | NT | NT | - | - |
| pQLLFRPa[2] | - | - | - | - | - | - | - | - | - | - | - | - | NT | NT | - | - |
| pQLFRPa[2] | - | - | - | - | - | - | - | - | - | - | - | - | NT | NT | - | - |

**Supplementary Table 3. DNA and Amino acid sequences of NeNaC2 Wild Type Organisms and Knock-out lines (deletion of five nucleotides).** The highlighted green text indicates the start codon, the highlighted yellow text the sgRNA region and the highlighted light blue text the stop codon (DNA). The highlighted magenta text indicates the N-termini, the highlighted navy-blue text indicates the TransMembrane Domains and the highlighted gray text indicates the C-termini (AA).

| | |
|---|---|
| DNA sequence of NeNaC2 WT | ATGTCGCTTGATATCTGCGATGCCTACATCCAACAGGAAACTGATATCGGAAAACTGTACTTTGTA<br>AATACACACATCTTGGACGCTGCAATAACTGGGTGCTTATCCCTGTATGATTTGAAGTTGATTGCTG<br>CAGTCATGTCGTGTCCAGTTGCACAGAGACATTTCGAAACGGAGGAGGATAAAAAAGATGAAGAC<br>GAGGATGACCGGGCCGAGGATCCTGTTGATGAGAATCCTGATGACACGATCACGGTGTCGCAGAT<br>GTGGCAAGACTTCCTGCACACCCTGACACTACACGGCTTTCGGTTTGTCTTCGAAAGAGGCCCTAC<br>AATTCGCAAGGTGTTATGGCTTGCTATTCTGCTGTTCGCCGTAGGGATGCTGATGATGCAGAGCAA<br>GAAAAGCATACAGAAGTACTTCGACCACCCGATAACGACGAGCGTGCAGGTCGAGTTTCTGGAAG<br>AGATCCAGTTTCCCGCAGTCACAATATGTAACTTTAACCTGTTTCCGTATTATCTTATAAACGGAAC<br>GATCGGCGAAAAGGTGATGTCAATTTTGGCGCCACAGAAATACATCGATAACAAGGAAGAGGTGC<br>TCTTCGCGCGCTCGCCGATACCGAACTTCCTGAATTACGCACGCAAACGGCGTAGGTCAACAGGCG<br>GAACGATAGTAACGGACGACATGCTGCAGAGCGAGAAAGACTTCGGCGAACTCGATGAAAAGTTC<br>GATTTTGCCGAGTTCGTGAGAACGCATGGTCATCGCATCGACCACATGATAAAAAAATGTCGCTGG<br>AAGTCGCAGCCCTGTGGTCCTGAAAACTTTACGGCGGTCATAACAGAATTCGGTCTTTGCTATACCT<br>TCAACTCAGGCATGAAAGGCCACCCTTTACTCAAGGTGCAGAGAGCAGGTGTAGACTACGCCCTTC<br>GGCTGCAGCTCAGCGTTCAGCAGGATCAGTATTATGGCTCCCTGCGCGATTCCTCAGGCTTCAAGG<br>TCATGGTACACGACCAGGAAGAGCCACCACTTATCAACGAGCTCGGCATTGCCATACAACCTGGCA<br>CGCACACGTTCTGCGGCTTGAGAAAAGAAGAGATGCATAATCTCCCAGCGCCGTTCAAAACCGCCT<br>GTCGAGACATGCAGCTAGAAGGCTTCAAGAAATACACCAAGTCAGCATGTCTTTTGAAATGTCGCG<br>CAGACTATGTGATGAAAATGTGCAAGTGTCGCTCTTATGACCTTAAAGGCCCCGCCCCGCCCTGTC<br>AGCCTAGGGAAGTTAAGAACTGCGTTTGGCCCGCAATGGAGATATTCCGCAATGAAAGTATCAACT<br>GCGAGTGTCCAGTCCCTTGTGAGATCACAAAGTACCAAACGCAATTATCTTATGCCCAGACCCCGG<br>CCAAACACTTCTCCGAGGTGCTGGCAAGAAGGAAACACATCAATAAGGATGTCATGAGGCACTAT<br>CTCAGGGATAATTTCTTAGAGCTCGATGTTTACTTCGAGGAGATGCAAGTGACGCTCATTCAGCAG<br>CGACAAGCATATGACCAGGAAAGCTTGTTTGGCGATATTGGTGGTCAAGTAGGGTTGTTTCTTGGA<br>GCAAGCATTCTTACTGTCCTCGAGTTCCTGGACTTGTTATGGAGAATACTCATTCACAAGTTCAAGA<br>AGAGAAAAAAACAGAAAAGTAAGGAATGTATAG |
| DNA sequence of NeNaC2 knock-out line | ATGTCGCTTGATATCTGCGATGCCTACATCCAACAGGAAACTGATATCGGAAAACTGTACTTTGTA<br>AATACACACATCTTGGACGCTGCAATAACTGGGTGCTTATCCCTGTATGATTTGAAGTTGATTGCTG<br>CAGTCATGTCGTGTCCAGTTGCACAGAGACATTTCGAAACGGAGGAGGATAAAAAAGATGAAGAC<br>GAGGATGACCGGGCCGAGGATCCTGTTGATGAGAATCCTGATGACACGATCACGGTGTCGCAGAT<br>GTGGCAAGACTTCCTGCACACCCTGACACTACACGGCTTTCGGTTTGTCTTCGAAAGAGGCCCTAC<br>AATTCGCAAGATGGCTTGCTATTCTGCTGTTCGCCGTAGGGATGCTGATGATGCAGAGCAAGAAAA<br>GCATACAGAAGTACTTCGACCACCCGATAACGACGAGCGTGCAGGTCGAGTTTCTGGAAGAGATC<br>CAGTTTCCCGCAGTCACAATATGTAACTTTAACCTGTTTCCGTATTATCTTATAAACGGAACGATCG<br>GCGAAAAGGTGATGTCAATTTTGGCGCCACAGAAATACATCGATAACAAGGAAGAGGTGCTCTTC<br>GCGCGCTCGCCGATACCGAACTTCCTGAATTACGCACGCAAACGGCGTAGGTCAACAGGCGGAAC<br>GATAGTAACGGACGACATGCTGCAGAGCGAGAAAGACTTCGGCGAACTCGATGAAAAGTTCGATT<br>TTGCCGAGTTCGTGAGAACGCATGGTCATCGCATCGACCACATGATAAAAAAATGTCGCTGGAAGT<br>CGCAGCCCTGTGGTCCTGAAAACTTTACGGCGGTCATAACAGAATTCGGTCTTTGCTATACCTTCAA<br>CTCAGGCATGAAAGGCCACCCTTTACTCAAGGTGCAGAGAGCAGGTGTAGACTACGCCCTTCGGCT<br>GCAGCTCAGCGTTCAGCAGGATCAGTATTATGGCTCCCTGCGCGATTCCTCAGGCTTCAAGGTCAT<br>GGTACACGACCAGGAAGAGCCACCACTTATCAACGAGCTCGGCATTGCCATACAACCTGGCACGC<br>ACACGTTCTGCGGCTTGAGAAAAGAAGAGATGCATAATCTCCCAGCGCCGTTCAAAACCGCCTGTC<br>GAGACATGCAGCTAGAAGGCTTCAAGAAATACACCAAGTCAGCATGTCTTTTGAAATGTCGCGCAG<br>ACTATGTGATGAAAATGTGCAAGTGTCGCTCTTATGACCTTAAAGGCCCCGCCCCGCCCTGTCAGC<br>CTAGGGAAGTTAAGAACTGCGTTTGGCCCGCAATGGAGATATTCCGCAATGAAAGTATCAACTGCG<br>AGTGTCCAGTCCCTTGTGAGATCACAAAGTACCAAACGCAATTATCTTATGCCCAGACCCCGGCCA<br>AACACTTCTCCGAGGTGCTGGCAAGAAGGAAACACATCAATAAGGATGTCATGAGGCACTATCTC<br>AGGGATAATTTCTTAGAGCTCGATGTTTACTTCGAGGAGATGCAAGTGACGCTCATTCAGCAGCGA<br>CAAGCATATGACCAGGAAAGCTTGTTTGGCGATATTGGTGGTCAAGTAGGGTTGTTTCTTGGAGCA<br>AGCATTCTTACTGTCCTCGAGTTCCTGGACTTGTTATGGAGAATACTCATTCACAAGTTCAAGAAGA<br>GAAAAAACAGAAAAGTAAGGAATGTATAG |
| Amino acid sequence of NeNaC2 WT | MSLDICDAYIQQETDIGKLYFVNTHILDAAITGCLSLYDLKLIAAVMSCPVAQRHFETEEDKKDEDEDD<br>RAEDPVDENPDDTITVSQMWQDFLHTLTLHGFRFVFERGPTIRKVLWLAILLFAVGMLMMQSKKSIQK<br>YFDHPITTSVQVEFLEEIQFPAVTICNFNLFPYYLINGTIGEKVMSILAPQKYIDNKEEVLFARSPIPNFLNY<br>ARKRRRSTGGTIVTDDMLQSEKDFGELDEKFDFAEFVRTHGHRIDHMIKKCRWKSQPCGPENFTAVITE<br>FGLCYTFNSGMKGHPLLKVQRAGVDYALRLQLSVQQDQYYGSLRDSSGFKVMVHDQEEPPLINELGIA<br>IQPGTHTFCGLRKEEMHNLPAPFKTACRDMQLEGFKKYTKSACLLKCRADYVMKMCKCRSYDLKGPA<br>PPCQPREVKNCVWPAMEIFRNESINCECPVPCEITKYQTQLSYAQTPAKHFSEVLARRKHINKDVMRHY<br>LRDNFLELDVYFEEMQVTLIQQRQAYDQESLFGDIGGQVGLFLGASILTVLEFLDLLWRILIHKFKKRKN<br>RKVRNV |

| | |
|---|---|
| Amino acid sequence of NeNaC2 knock-out line | MSLDICDAYIQQETDIGKLYFVNTHILDAAITGCLSLYDLKLIAAVMSCPVAQRHFETEEDKKDEDEDDRAEDPVDENPDDTITVSQMWQDFLHTLTLHGFRFVFERGPTIRKVAIMACYSAVRRRDADDAEQEKHTEVLRPPDNDERAGRVSGRDPVSRSHNM* |

**Supplementary Table 4. Accession numbers of the 29 NeNaCs from different genomic databases.** NVE models (available at https://figshare.com/articles/dataset/Nematostella_vectensis_transcriptome_and_gene_models_v2_0/807696), UniProt and GenBank. *NeNaC3 has two NVE models.

| NeNaCS | NVE number | UniProtKB | GenBank Number |
|---|---|---|---|
| 1 | NVE20900 | A7RI82 | XP_031568943.1 |
| 2 | NVE13023 | A7RGQ6 | XP_032222736.1 |
| 3* | NVE21425 | A7S2F2 | XP_032239479.1 |
| | NVE9704 | | |
| 4 | NVE574 | A7SA77 | XP_032236192.1 |
| 5 | NVE578 | A7SA83 | XP_032236199.1 |
| 6 | NVE1283 | A7RL38 | XP_032220870.1 |
| 7 | No | A7SH56 | XP_001629025.1 |
| 8 | No | A7SJB5 | XP_032232203.1 |
| 9 | No | A7SST7 | XP_032228649.1 |
| 10 | NVE25684 | A7S8S3 | XP_032236827.1 |
| 11 | NVE6385 | A7SJJ5 | XP_032232107.1 |
| 12 | NVE23003 | A7S4N0 | XP_032238530.1 |
| 13 | No | A7SBZ4 | XP_032232203.1 |
| 14 | NVE15642 | A7SX80 | XP_032226792.1 |
| 15 | NVE10030 | A7SPS3 | XP_032229849.1 |
| 16 | NVE11038 | A7SRD9 | XP_032229236.1 |
| 17 | No | | XP_032233690.1 |
| 18 | NVE3951 | A7SFR1 | XP_032233718.1 |
| 19 | NVE15634 | A7SX71 | XP_001623796.2 |
| 20 | NVE3950 | A7SFR0 | XP_032233720.1 |
| 21 | NVE3518 | A7FS65 | XP_032233957.1 |
| 22 | NVE16828,16829 | A7RWI8 | XP_001636193.2 |
| 23 | NVE12626 | A7STM2 | XP_001625049.2 |
| 24 | NVE20901 | A7RI83 | XP_032222096.1 |
| 25 | NVE24072 | A7RJS8 | XP_032221585.1 |
| 26 | NVE3545 | A7SF92 | XP_032233942.1 |
| 27 | | A7SZH3 | XP_032225907.1 |
| 28 | | A7SZH6 | XP_032224488.1 |
| 29 | | A7SP23 | XP_032230128.1 |

**Supplementary Table 5. Sequences that were removed for the final alignment to construct the molecular phylogenetic tree due to extreme distances or fragmentation.** The sequence of HyNaC1 was not included in the alignment.

| Sequences |
|---|
| >Aqueenslandica_XP_011405346.1 |
| >Aqueenslandica_XP_011405345.1 |
| >Pdamicor00010574 |
| >Pdamicor00001790 |
| >Pdamicor00000909 |
| >Actiniatenebrosa17_XP_031566216.1 |
| >Actiniatenebrosa18_XP_031564163.1 |
| >Actiniatenebrosa19_XP_031561615.1 |
| >Amplexidiscus_fenestrater14 |
| >Stylophorapistillata11251 |
| >Stylophorapistillata3268 |
| >Stylophorapistillata14084(1) |
| >Stylophorapistillata104 |
| >Stylophorapistillata4304 |
| >Stylophorapistillata2660 |
| >Mleidy19430 |
| >Mleidy125633 |
| >Mleidy126004 |
| >Mleidy187866 |
| >Mleidy184955 |
| >Scolantuscallimorphus31308 |
| >Scolantuscallimorphus45179 |
| >Scolantuscallimorphus6332 |
| |

**Supplementary Table 6. Primers for the generation of amplicons for the ISH experiment.**

| Gene | Forward | Reverse |
|---|---|---|
| NeNaC1 | 5’ATGGCAAAGAGTTGAGCGTGGCCGAGTC | 5’CCGAAAGACCTTGCATTTTGACTGGCGT |
| NeNaC2 | 5’CACATCTTGGACGCTGCAATAACTGGGT | 5’ACTGATCCTGCTGAACGCTGAGCTGCAG |
| NeNaC3 | 5’GGTGGGCCTTGTCAATCAATACAACAGC | 5’ATATTCCGTTTCTGCATCTCAGGGGTGT |
| NeNaC4 | 5’TCAACATGGATGCTATCGTACAGAAGAG | 5’AAGCTCATCGGGTGACAAACCCGTCAGA |
| NeNaC5 | 5’CGAGAAAGTCTTCTCGAAGCCTTCAGCT | 5’GCTTTACCACCATGTCCGAGTAACACTG |
| NeNaC6 | 5’TCCACTTTTTAATTCACCCTGGCTCGCC | 5’CTTGATAGGTGGGAATCCAATAATCCTG |
| NeNaC7 | 5’TCGCCGAGTCACGTGGCTTCTACTTCTG | 5’AAAGGGCTACGCACCCCTCCATTGAGTA |
| NeNaC8 | 5’ATGATATTCCTGGCAGGTCTGGGAATGG | 5’TATGGTCCTCCTGAAAGAGTAATGTGTG |
| NeNaC9 | 5’GTTGGAGGCGCCGCATCTGGTTCCTTTT | 5’CGCGAGGTTTGGGGCAATCACATAACTC |
| NeNaC10 | 5’ATGAACTTGTTCCAGACGAAAGTTAAAC | 5’CTAGTCATCCTGGATTGCTGCCCGGGCT |
| NeNaC11 | 5’CAAGGGCGTCGAGAGTCCAGAAGAAGTC | 5’TTCACGAATGGTAGCGAAGACTGGCCGA |
| NeNaC12 | 5’TTGATTCATACTCCGTAACTCGTCGAGT | 5’CATCTTTCATTGGTGGCATGCCTAACAA |
| NeNaC14 | 5’GCTGTGACCATCTGCAACTTGAACATGA | 5’ACAACCCTGTACGGAATACTTGAGGCTT |
| NeNaC15 | 5’GGACTGGGAATGGTGGTTCATCAAGGCG | 5’GTTTGTTCGCCAGACAGGAGTTCATACA |
| NeNaC16 | 5’GTGGATATTGTTGCTGCTTGTCGCATTT | 5’GCCGGTGGGCAGTTACAAGAAGACATGC |
| NeNaC21 | 5’GCACGGTTTTGCACGACTCGTTGAATCC | 5’GCGTTCTCTGTTAGACCCGATCATTGCC |
| NeNaC22 | 5’CAGCGGCAGCGCTTGTAACCCAATTGAC | 5’TGACCTCGTAGATATTCTCCTCACACGG |
| NeNaC23 | 5’GACCCCTGGAACAAGGATGAGAAACTTA | 5’AACACCAAATGGTTCGGGTAAACTGATC |
| NeNaC24 | 5’CGATCTGCTGCAAAACGAGACAAGAAACA | 5’TGGAGGATGTATTTAGTCAGGCTCTCCA |
| NeNaC25 | 5’TCCCTAAAGACGGCCTCGACTTTCCCGTG | 5’CGTAGATTTGTTCTTTGCACGGGACTGT |

## SUPPLEMENTARY FIGURES

**Supplementary Figure 1. Extended Maximum Likelihood tree with the marine sponge *Amphimedon queenslandica* as outgroup.** The tree was inferred from an alignment of 287 DEG/ENaC protein sequences under the WAG+FO+I+G4 model. The branch support was estimated with SH-aLRT, abayes test and ultrafast bootstrap shown on the internal nodes. As the tree is very large, it is shown in the next page.

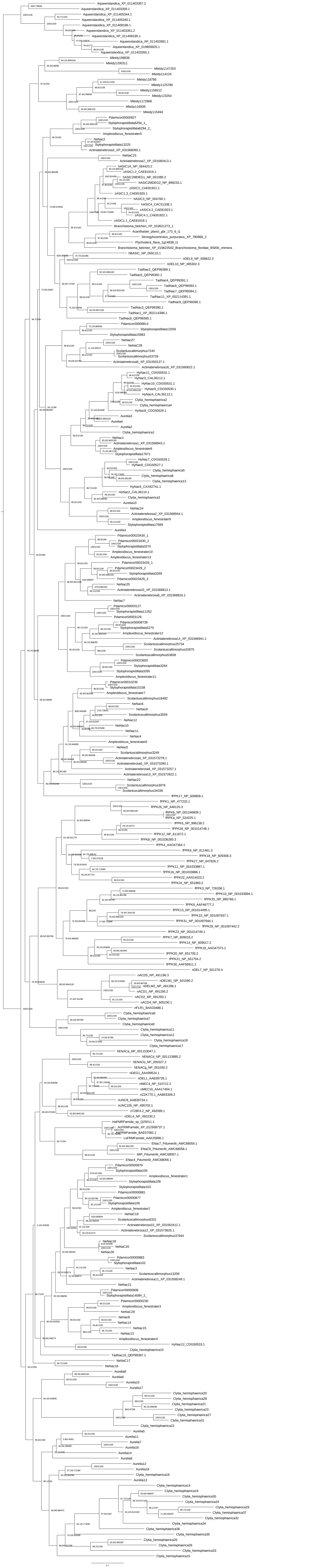

**Supplementary Figure 2. Extended Bayesian phylogenetic tree with the marine sponge *Amphimedon queenslandica* as outgroup.** The tree was inferred from an alignment of 287 DEG/ENaC protein sequences under the GTR20 model. Bayesian posterior probabilities (PP, in percentage) are shown on the internal nodes. As the tree is very large, it is shown in the next page.

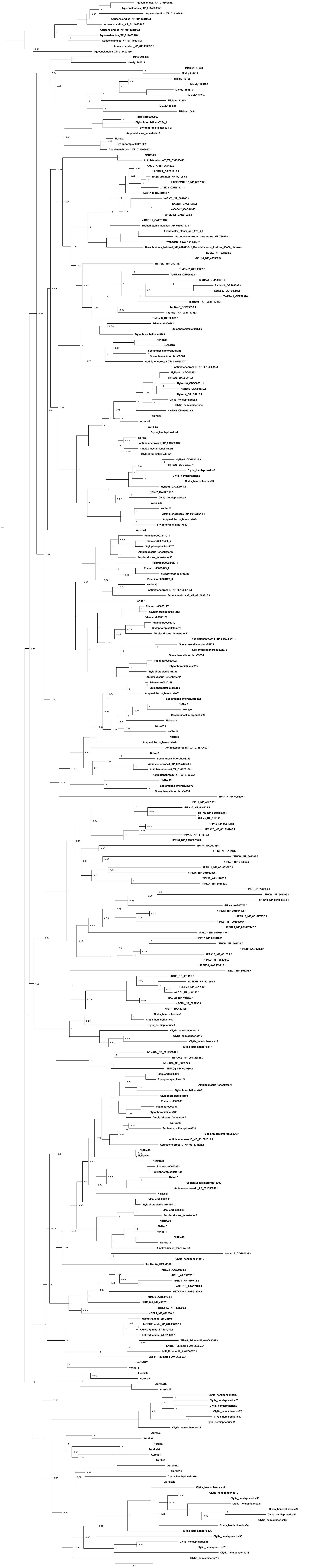

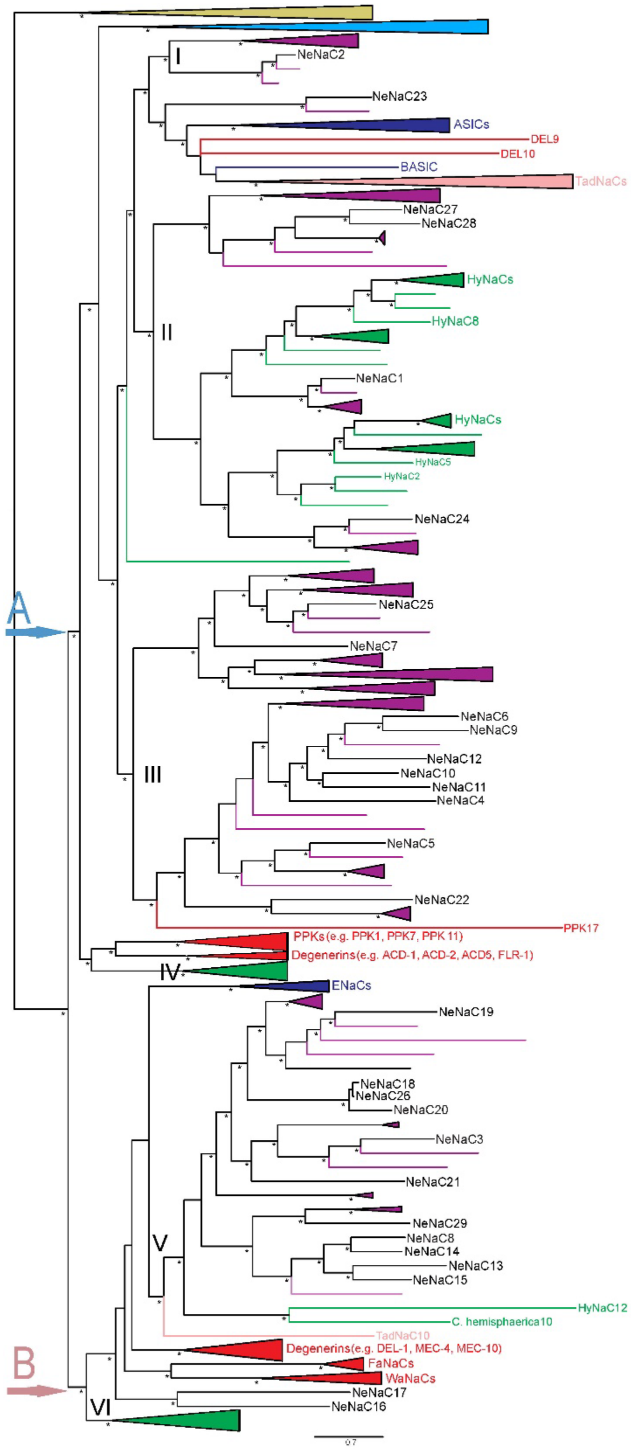


**Supplementary Figure 3. Collapsed Bayesian phylogenetic tree considering marine sponge *Amphimedon queenslandica* as outgroup.** The asterisks below branches denote a posterior probability >65.

**Supplementary Figure 4. Extended Maximum Likelihood tree with the comb jelly *Mnemiopsis leidyi* as outgroup.** The tree was inferred from an alignment of 287 DEG/ENaC protein sequences under the WAG+FO+I+G4 model. The branch support was estimated with SH-aLRT and ultrafast bootstrap shown on the internal nodes. As the tree is very large, it is shown in the next page.

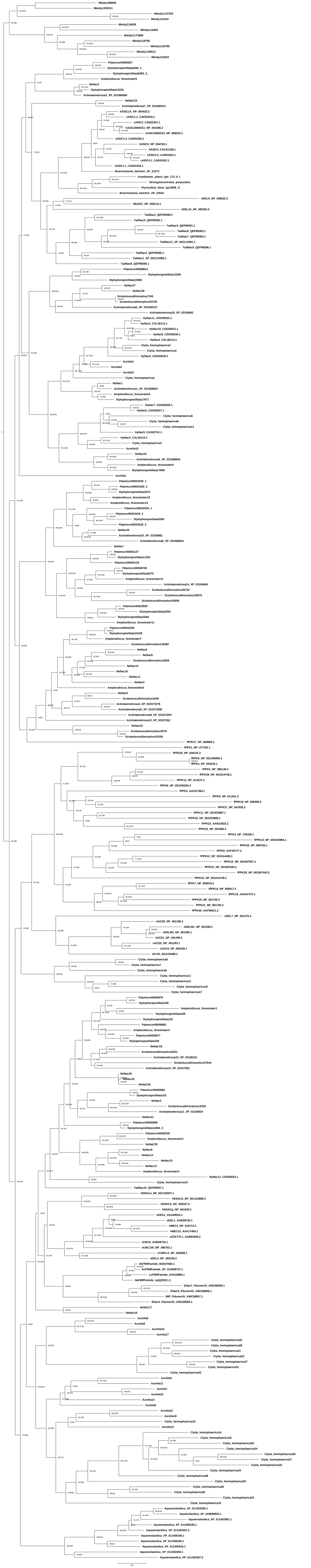

**Supplementary Figure 5. Extended Bayesian phylogenetic tree with the comb jelly *Mnemiopsis leidyi* as outgroup.** The tree was inferred from an alignment of 287 DEG/ENaC protein sequences under the GTR20 model. Bayesian posterior probabilities (PP, in percentage) are shown on the internal nodes. As the tree is very large, it is shown in the next page.

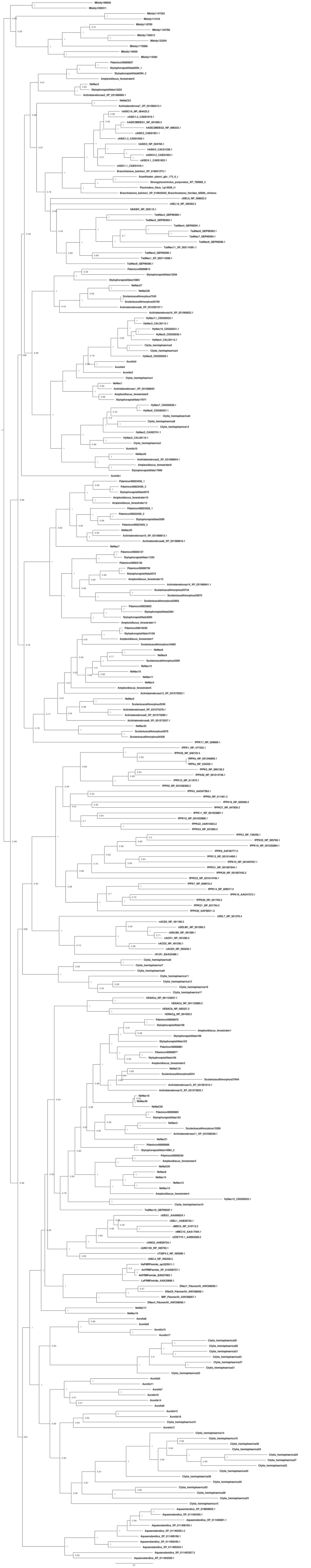

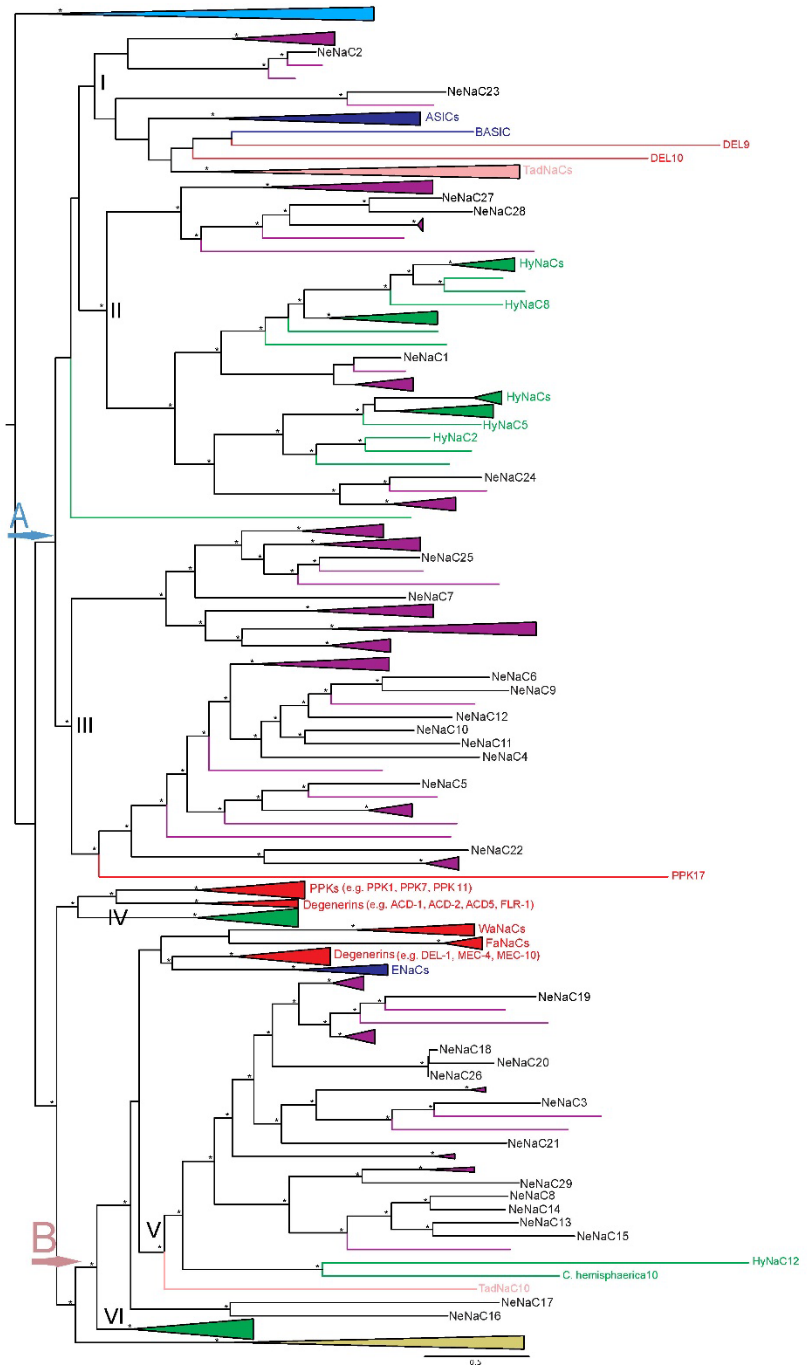


**Supplementary Figure 6. Collapsed Maximum Likelihood tree considering comb jelly *Mnemiopsis leidyi* as outgroup.** The asterisks above branches denote a bootstrap support >0.65.

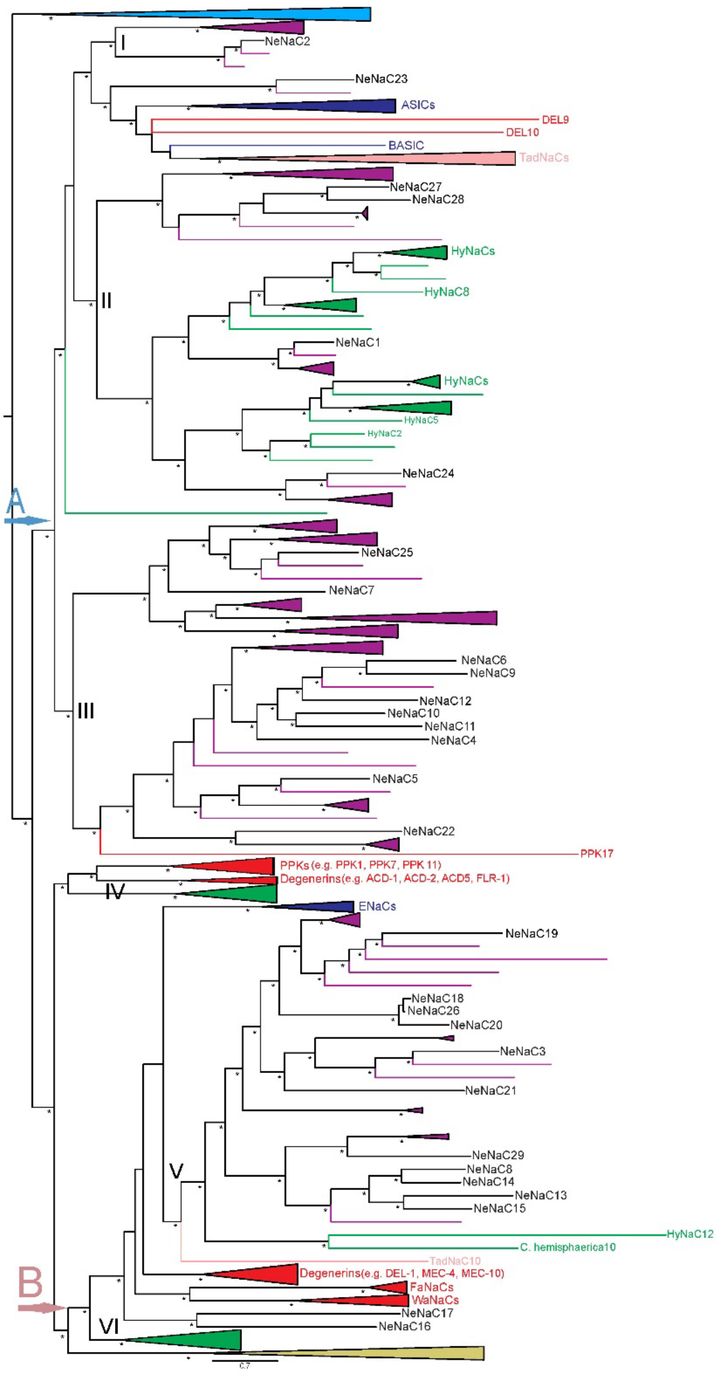


**Supplementary Figure 7. Collapsed Bayesian phylogenetic tree considering comb jelly Mnemiopsis leidyi as outgroup.** The asterisks below branches denote a posterior probability >0.65.

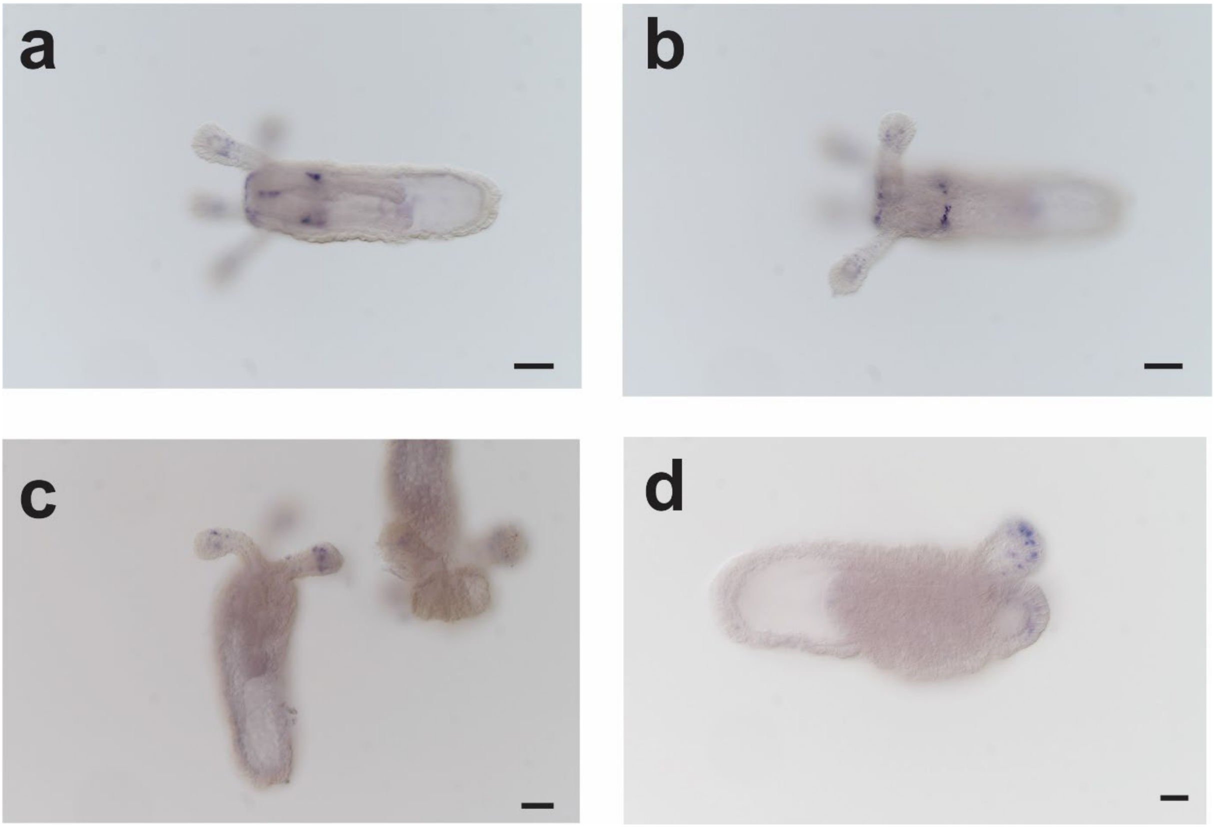


**Supplementary Figure 8. In situ hybridization of selected NeNaC channel transcripts at the primary polyp stage (9 dpf).** The expression is noticeable by the blue stain (NBT/BCIP crystals). **a** NeNaC1; **b** NeNaC1; **c** NeNaC2; **d** NeNaC2. Scale bars: 100 µm.

```
NeNaC2   ------------------------MSLDICDAYIQQETDIGKLYFVNTHILDAAITGCLSLYDLKLIAAVMSCPVAQRHF
NeNaC23  --------------------------------------MSNAPDYRNFIEALHQTLNNPNNRVAVDNRGYALESPLDFYK
NeNaC1   --------MKENDKDEDGGDVKIIIKGSDDKAPQGAGSIYAFCNHCKKDISVVYIKSENNGKELSVAESLTNKWPRSEFP
NeNaC24  -------------------------------------------MALCGCCASKPSPTNSASEDESTGPESKEEILLANQN
NeNaC7   --------------------------------------------------------------------------------
NeNaC6   --------------------------------------------------------------------------------
NeNaC9   --------------------------------------------------------------------------------
NeNaC12  --------------------------------------------------------------------------------
NeNaC10  --------------------------------------------------------------------------------
NeNaC11  --------------------------------------------------------------------------------
NeNaC5   --------------------------------------------------------------------------------
NeNaC3   --------------------------------------------------------------------------------
NeNaC8   --------------------------------------------------------------------------------
NeNaC14  --------------------------------------------------------------------------------
NeNaC15  --------------------------------------------------------------------------------

                                                                   TMD1
NeNaC2   ETEEDKKDEDEDDRAEDPVDENPDDTITVSQMWQDFLHTLTLHGFRFVFERG-PTIRKVLWLAILLFAVGMLMMQSKKSI
NeNaC23  NNWVGDGESVDQKKAQEVDPWNKDEKLTISQHFASLCSSTTMHGISNVFDPSSATLRKGIWSVVFLCSFIFCAYEIGNNV
NeNaC1   ENDYDKCLKDNDYGGCKKLLDGRKKRAKVKELWENFLGGCTLHGFHYCFAGN-PPLRRLIWSLLLLGAFAMFFEKCTESF
NeNaC24  GNASTYENIRKRSAAKRDKKHAEYGKRKMREVFAYYINHCTLHGFHYIFETK-SLFRKIAWFSVLAIAGGFFFKEIKTST
NeNaC7   -------MTSANDDLTNDSRGETNRVGQVRPLWRDFLSRTTLHGTQYACVTK-PLIRRVTWLLLLLGMVGYFGYLFYGNL
NeNaC6   -----MESFNQNTKTDIKDCKEDEKREPQPTMLQDFAGYTTLHGFHFLIHPG-SPFRRFIWLLMLLGCWVALFYQLYNSV
NeNaC9   ------MDPPEERNKEDAREKEHKEENETWNMIREFAGYTTLHGFHFLVDSS-SRWRRRIWFLLLLFCCAMFIYQFVISV
NeNaC12  ----MSYNCKVEDSSDINSNNEYRGGSKTRQLIKEFSGYTTLHGFHFLVDSY-SVTRRVVWTCFIVISLGFLLYQLVNGI
NeNaC10  -MNLFQTKVKPLSRDEAKSDSEKEIEEQRKTLIKNFSSYTTLHGFHFLLDSS-PMPRRVLWTALVVFGLVFFFIQLVMSY
NeNaC11  --MKKDAFETSNQTNFSVFDENEDIRQKRKKLISEFSGYTTLHGLHFLIDSG-SLFRKVFWMILLLVMFTCFFIQLVESY
NeNaC5   ---------MTTVKVANHENPSESVEVPRESLLEAFSSYTTLHGFHFALSST-NRVRQIIWILLLILTSVVLIYQLAYST
NeNaC3   ---------MLKLPCNKEMLRNADPKETALEQINQFLQETTAHGFGRLGATA-GSKWRIYWVMFCLAAYCVFAWQLVGLV
NeNaC8   -------------------METLKPHPSVRAIFKDFSDRTSCHGIGQIGGSQ-SVMWRVSWLMIFLAGLGMVLYQGLTLL
NeNaC14  ---------------------MSRSGPSVRALLRDFSDRTSCHGIGQINGSH-SPTWRIFWLLTFLAGLGMVLFQCITLL
NeNaC15  --------------------MADQPRPSVRALVRDFVDRTTCHGIGQINGSQ-SPLWRVFWVVTFVAGLGMVVHQGVTLF

                                 I
NeNaC2   QKYFDHPITTSVQVEFLE-EIQFPAVTICNFNLFPYYLINGTIGEKVMSI-------------LAPQKYIDNKEEVLFAR
NeNaC23  RYYLTKPVNTVFKIDYVD-EIKFPAVTICNNNPIRKSWAATT-------------------------PY-----------
NeNaC1   INFFDYPFTTTTLLVYDK-RLPFPAISMCNYNDARMSKMNGTLMNEIFVA-------------SKLE-------------
NeNaC24  TQYFKYPFSIMSTVEYPR-TLVFPAVTICDFHDIRQTLVSNAKGEQAIN-------------------------------
NeNaC7   KRYHSHPVEVTVEIETPNDGIGFPAVSVCTNNKYMKSKINMLRN----------------------HSYFHKLGLDIPEC
NeNaC6   DRLLAHRIVMSRGVEQPD-EIDFPAITFCNQNIMRMSKINGTEA----------------------QKYLDELDDVFR--
NeNaC9   NRLRAYKVVFSRGAEQPG-EVDFPAVTICNKNMLRKSLILNTSA----------------------QIYLDEQDADFK--
NeNaC12  KNYNDRGIIMSRSVEEPN-EVDFPAVTICNQNIMKKSLIIGTDA----------------------QRYLDEMNYIKA--
NeNaC10  GKLRARESILAKGVERPM-NVLYPAVTICNQNMMRKSRITGTAA----------------------QRYLDQLDHIKA--
NeNaC11  KRLKEYGSNLSKGVESPE-EVTVPAITICNQNMMRKSLVMGTDA----------------------QKYLDGQDIMKI--
NeNaC5   QRVLEYASMVQVETRNED-SITFPAISICSNNMMQKSKILGKDA----------------------QRYLDLLDQKKE--
NeNaC3   NQYNSKPIKTRTQLKHAQ-KLDFPVVTICNMNVLRASRLPPKLRTKFDEIINNTKKTSSRKSSNSRNAFVDPQDLSFEET
NeNaC8   DTYLNKPTATAVDITYSE-VTNFPSVTICNMNMIKKSQLQHFPQV--KRLVDTFNNMTSSNSSLNSSAFMDTNREKAEKD
NeNaC14  GIYLDKPTATSVDVTYDE-VTNFPAVTICNLNMIKKKNLANFTQS--KKIFDDFEAFVSSNSSMDSSAFLGSKMETVLKD
NeNaC15  GTFLDRPTSTTIDMTYAP-AMDFPAVTICNLNAIRKDHLSQFPDA--DVLLKGFSEAST----PSVTVFLGKDVESFIKQ

                                                                              II
NeNaC2   SPIPNFLNYAR----KRRRSTGGTIVTDDMLQS----EKD--FGELDEKFDFAEFVRTHGHRIDHM----IKKCRWKS--
NeNaC23  --LPVIMAYNA----NP----GEEAIPIN--------WD----AYNWTGFGFDKLMSSAAHLASEM----IHTCKWKG--
NeNaC1   ---------------------GRNT-------------SH--LQSQLTGELMQRTLKEAAHRLPDM----IKECSWQK--
NeNaC24  ------------------------------------------ISSSE---KLETLARKTYKSFNET----LISCSLRRGV
NeNaC7   AALQNVSGNMTCGQALMCAIVGKYGYIINERCKWALEKIRKIINDSDYAFDIEKFTLKYGHDIKALLT--PRFCTFRG--
NeNaC6   --------------------------------------KD--IKKENVSYDAETFVNKYGHDWENMFENIPYSCMFQR--
NeNaC9   --------------------------------------RS--LQQSNISFDAEEFAKKYGHNITNMLNR-EKGCTFKM--
NeNaC12  --------------------------------D----LGL--VNSTNERLDAEDFVRKYGHTLGEM----MYGCEFKD--
NeNaC10  --------------------------------S----LSR--VNRTNERFETEEMVRLYGHNITDM----LWECNFMN--
NeNaC11  --------------------------------K----LGA--AQVSNESFEVDKMVREKGHLLESM----LFECSFAG--
NeNaC5   --------------------------------D----QWDAISQSFSPDFDIEKAVHQYGLNLSLA----MKSCHYGR--
NeNaC3   K-------------------------------K-----IEILHA--VTTHDNYRELVSAAHQLEDI----LLSCNFNG--
NeNaC8   RL-SLNTKNS-------KNKVILNNVNIDMQRY----IEDKIVQ--YLSMSDTTKLMKAGHVFREL----VFRCVWNG--
NeNaC14  RL-SMDSNDG-------SNSVSLDDTSMDTELY----VEDMLIR--HMAMVDDKDLIEAGHEFDEL----VFRCVWNG--
NeNaC15  HS------------------EAGPNVTLDPELA----FKDAMVE--IFAQSELKKLQMAGHGFEEL----VLGCTWNN--
```

```
               II                       I
NeNaC2   ---QPCGPE---NFTAV----ITEFG-LCYTFNSGMKGH-----PLLKVQRAGVDYALRLQLSVQQDQYYGSLR-DSSGF
NeNaC23  ---IYCSAA---NFTLD----ATFLG-GCYTFNIDQR---------LMVTGTGMANALHLVLNIQQNEYIGNVR-SGAGF
NeNaC1   --HGKCSWK---NFTSF----KSADGDTCYTFNSGRK-D-----PILSMSNVGEENGLRLVIDTQHSEYYYDVK-N-AGF
NeNaC24  RGARPFNIH---DFKVF----FTAKGQTCYTFNAAMDGK-----KLLEVDNVGPKFGLEIYLNAQHWWFKDDVR-E-SGF
NeNaC7   ---KPCNEE---DFVPV----ITSTS-LCWTFNSGFRGSHGNPAPRKQVTFSGVDFGLTVLLNTRVDENTIGT--SSEGV
NeNaC6   --FFICSAK---NFTSF----LSFTRGLCYTYNSGVNRS-----YVQRVSEAGRNNRLEFHLEAHPEEYYGPYSYEGIGF
NeNaC9   --LYPCSGE---NFTSF----FSFTRGWCYTFNAGA--N-----YIQRVSVAGRETRLKLYLDAKSHEYYGPFSYDGVGF
NeNaC12  ---RRCTAQ---DFIVS----TSFMRGLCYTFNSGRDNS-----SVRRIATPGRLESLILRLNAQPEEYYGAYSYENVGF
NeNaC10  ---KPCSHK---DFAMRY---TSYSRGLCYTFNSGANGS-----PIGQATTSGTRTSLSLRLNAESDEYYGPFSYDATGF
NeNaC11  ---TTCTPE---NFTTS----LSFTRGLCYTFNSGTNNT-----PVFTARAADIRMAFSAMLFSQPEEHYGPFSHRATGF
NeNaC5   --YLKCNPS---HFTTF----KEFRYGLCYTFNSGKRES-----AF-ISHDTGPTSGLSITLDAQPEEYYSLYSSTGTGF
NeNaC3   ---VNCRNSNDPTIPTSWTQTWNDNFGNCYMFNPAQTHN-GEKVDPYSSSIPGESNGLTLQLNIEQNEYLEGIT-EVAGI
NeNaC8   ---FVCNKG---DFLKYWRPFWHWRYGNCYTFNQGVDVN-GTELPSLASSKPGPMYGLTLDLFIDQEQYIIPLS-QEAGV
NeNaC14  ---FTCNKG---GFMKFWRRFWHWRYGNCYIFNQGVDEN-GTLLAHLTSSKPGPMYGLTLDLFIDQEQYLIPLS-QEAGV
NeNaC15  ---IKCNKG---DFLKYWRPFWNFRYGNCYTFNQGMSEK-GVAIKPLTLSNTGPNYGLTLDLFINQEQYIAPYT-QEAGV

                                                 III                          IV  V
NeNaC2   KVMVHDQEEPPLINELGIAIQPGTHTFCGLRKEEMHNLPAPFKTACRDMQ--L----------EGFKKYTKSACLLKCRA
NeNaC23  RLLFREKHEPPSTDRFVIALQPGTQTLIPLTMKKLISLPEPF-GVCQEKNN-L----------KMFDKYSVTACEFECRA
NeNaC1   KVILHDQGETPVK-MQGLSVSPGFTSYMELKRTKVTNLPFPYKTMCGMPE--L----------KYFNSYSKSKCFLDKLT
NeNaC24  RFILHQQDDLPLT-REGFRVSSGYVTYVDMRLEKVKNLPYPFSSDCDGRG--L----------DLYPKYSRNNCYMESLT
NeNaC7   RAVVHEPGEYFSV-DHGVNVMPGAHAAILVHAQKTTTLPLPYKSNCTESK-------------PGLRLYSMEGCVALCAS
NeNaC6   KIAVHDQSYVPNMDQEGYDITAGFYTNVRVKRYKEKSLPHPYKTNCGERK--L----------EYYERYSGSACILECQA
NeNaC9   KIAVHDQNDVPDMDNGGYDISPGYLTTISVKRFKEVSLPPPFPTKCGSRT--L----------EHYERYSTKGCEYECFA
NeNaC12  VLAVHDQAEPPDMELNAYDIPPGFTTNLRIRRFKENSLPEPYPTKCGSRN--L----------SLYKRYSRKACMQECYA
NeNaC10  KLAVHDQNEIPNMDEDAFDTSPGFLTNIRIRREKEINLPSPYRSECGSRD--L----------SNAPKYSMSGCIYECYS
NeNaC11  KIAIHDQSETPDIDLESYDLSPGFATNIRLIREKAKYLPAPYSSNCSSKR--G----------IDGGTYSETGCLTRCYN
NeNaC5   RVIVHDQSEFPWVEKHGWEIPPGFSTNVRLARKEISSLESPYNSNCSRDT--N--------------YASQSYCLVQCYS
NeNaC3   KVSISDQGVLPFPGQQGIRIMPGQSTGIQMTKLQTGRIDPFKNRSCENSN-EMSDKN---LFFGYNMRYSKMACKYSCLN
NeNaC8   KVLLSDQRNIPFPFTHGFTVQPGVSASAGIRQLVIKRIDPFSNGSCYSG-NGLEANSIYHKY--KGMRYSVQGCMSSCLA
NeNaC14  KVLLSDQRNVPFPFTDGFSVQPGVSASVGIRKLVINRIDPFNNGSCYSG-DGLEKENIYSKY--KSLKYSVQGCMSSCLA
NeNaC15  RILLSDQNQIPFPDSDGFTVSPSSSSAVGIKKIFITRIDPFNNGSCYKVTKGLEEGSIYKSVFSHDMGYSVQGCMNSCLA

               VI VII              VII      VI             V    IV   III
NeNaC2   DYVMKMCKCRSY---DL--KGPAPPC--QPREVKNCVWPAMEIFRNESINC--ECPVPCEITKYQTQLSYAQTPAKHF--
NeNaC23  RLGGKLCGCREMYPSSI--KTEIPVC--LPKAYRDCLNPLLVEISVN-NLC-RGCKNPCNKVTFVPRMSYSQYPANHI--
NeNaC1   QYVVTLCGCRDWFMPG---EGKIPVC--DYETAASCMWKAWAYFEEN-KLD--QCPVACNSVEYSAQLSYARFPAN----
NeNaC24  KYILQQCKCRAWFMAD---VINTSTC--SIKEALDCMWPAWEDFDNA-YNY--TCPVDCEERVYKTRLSSALFLPQKLLP
NeNaC7   QELTRRCGCRPVGLPY---VDAASVC--SFKH-ETCAMDTFGSFDAQ--RC--MCNNACHRTMYNAKVSYARFPDQYIIR
NeNaC6   REFVRKTKCRIIGFPPIKVIRDVPFC--SVLKIETGLTYMYYNWNTN--QC--DCPKPCVEISYSAQMSLLQYPTPSLVR
NeNaC9   KDFVRKFKCKTLGMAPIKEIRNASFC--PVSVVYAALMDFHKNWHLE--LC--DCPKPCETVNYNAQLSTAHYPAPSLLD
NeNaC12  RLIITHCGCRLSGMPPMKDVIEAPFC--TSKEYLDCQLMMPVLLKPS--KC--DCPKRCQHIHYSVQPSLAHYPSKSVIK
NeNaC10  KIIADKCKCRVLGMALN-------------------------VELNPA--MC--DCPKPCRALHYKIQLSLAYFPSDHLWD
NeNaC11  NLMTSQCQCKILGHESDYK-NITGFC--STYQLKACVYEAWMVLRPQ--NC--DCPKPCTSLKYKAQISTSYFPSESLWG
NeNaC5   DMVVKRCGCHMLGMTEE--TGHTPWC--SPQQIKACVYTTSRRFQPN--MC--SCPVRCSRVEFDVQLSSLYYPPDNFWE
NeNaC3   AKTIERCGCTDYNTPELQ-KRNISLCNRLNNAIIDCLNKAYDTFEDG--SCDRECPPSCSEVSFDLTISSAKWPAKSYEK
NeNaC8   NNQFKVCNCTEGKFRA-K-GRP---C--MTEPEVKCLNNISKKYENGSLGCSKSCPQPCTHYSFRRTISQTQWSD-SYEK
NeNaC14  NSEFSICNCTEGKFRV-K-GRP---C--MSESEVKCLNTVNKMYEKGTLGCTRKCPQPCSHFSFRRTISQSQWSE-SYEQ
NeNaC15  NKQREMCNCTEGRFDMMT-GLI---C--QQIEAWRCLNRVNMMYQEGGLKCLEKCPQPCTQNVFSRSMSHAHWAE-EYKK

                                                                 *      TMD2
NeNaC2   ----SEVLARRKHI----------NKDVMRHYLRDNFLELDVYFEEMQVTLIQQRQAYDQESLFGDIGGQVGLFLGASIL
NeNaC23  ----ADSMAM--------------SMNTTRDFVRDNFLEVEIYFEDIMVEIIEQQEAFSLTSLVGIIGGTLGVFIGASII
NeNaC1   --NYAKMLAKEYGLK--------GSDEENRQYLRDNLIEIKIYYEDLTYFDVQQVPSYDLYSLLGDVGGQIGLFLGASLL
NeNaC24  LTKKYKFLMRPKGIP--------NDTEGAVDFILENYSVINLFFDELRLDTIQQTPAYGFFRLVGDVGGQLGLVLGASVI
NeNaC7   FIQETT------------------SYNYSAEYFRRNLVLVQVGMESLSYEHHRQVPAFPVESLLGAVGGHLGLLLGCSVL
NeNaC6   EIRKSF----------NDT----E---DYINNMRANSVIVSIFYETLLTDVFEEKNDYDISRFGSDLGGNLGLFLGCSLL
NeNaC9   ELSKFPL----EGFVNKSK----E---EYVKFVRDNIVVVEVFYETLLTDVLKEERDYDFNMFASDLGGILGLYLGTSLL
NeNaC12  ELLPSLN----MTEVNSTT----ERINEVNRIIREGHAIIRVFYETLRTEIIKEKPQYTLATLTSDMGGSMGLFLGCSVL
NeNaC10  SIFPVLLNFTELVKVNTTGKSQDEVLLQIQEALRKQIAQVQIYYETLLTDVLEEKPAYGISEFGSDVGGNMGLFLGCSLL
NeNaC11  SLIPFLG-QSSLPFVNLTGKTLEQATAEAQVNVRKSVCMVNVFFETLVTDILEEKPSYDLTMFGADLGGTMGLFLGCSIL
NeNaC5   TISNERS----LTIYAND-------TKKFQEWYRRRVIQLNVFYKELTTEVRKEKEAYTISDLAGDFGGNMGLFLGCSIL
NeNaC3   TVLKTLQT----EYG---------INMTK-EEVFENIAQVHVYYGELDYLLVQETLAYTFMSLLSDIGGQMGMWIGISAL
NeNaC8   TFQRLVRK-SDRGFA---------NKMNDASILRKNFLRVKLYYEELNRETITYSLSYPVENLLGDVGGQLGLWIGVSVI
NeNaC14  TFQKMVMK-SDKGFD---------KKMRDASVLRQNFLRVKLFYEELNTEAITYSRSYTTESFLGDVGGQLGLWIGVSVI
NeNaC15  ALSKFIPN-NGNG-----------SSVDPNELISHNILRVKIYFEELNMETITYKRNYPVESFLGDVGGQLGLWIGVSVI
```

```
          TMD2
NeNaC2  TVLEFLDLLWRILIHKFKKRKNRKVRNV 559
NeNaC23 TVSEFMEFLILIPFRR 494
NeNaC1  TVVEYLDLLGMVAYTSFKYRNG 532
NeNaC24 TIVEIIDLVIMYSIYWIKSKTVPRAATSG---T 506
NeNaC7  TVFEFIDFFIVALASMLRTNVTPNMDRET---KNL 503
NeNaC6  TLVEFFDLGIRWCLGRKDKVQRS 461
NeNaC9  TIAEFLDLGIRWCLRRRSDRSQVRSQAWV---K 470
NeNaC12 TICEFIDLFIQICLERRKRNEVINQK 471
NeNaC10 TFCEFIDLVFMLCLHRHRLRKEAK--------ERQARAAIQDD 470
NeNaC11 TICEFIDLVIILVANGWRKGKARVIDVKE---KPEARP 488
NeNaC5  TIAEFIDLLVVYLVHRHKKRTAKIQ 458
NeNaC3  TCAELVELVCVILANMS---NRSKKIVHISSNIGCFRPAKLGGVEAIEKVGGVKTIEQEVQGGVGIIEQEVQGAVGTIEQ
NeNaC8  TCAEFLKLLVDLAWYLASKMSGKTKTQVQDLNMQ 522
NeNaC14 TCAEFFKLLIDVVWYLARKVHGGPKKTVRDLNMN 520
NeNaC15 TCAEFAKLLIDLVLCVAKKMNRSDKVQSVCIGRGN 510

NeNaC3  EVQGGVGTIEQEMQGRVGTIEQ 578
```

**Supplementary Figure 9. Sequence alignment of all NeNaCs expressed in *Xenopus laevis* oocytes.** The 15 NeNaCs are shown in the same order as in Fig. 1, putting related NeNaCs together. The conserved N-terminal HG motif, the conserved W residue in TMD1, the selectivity filter in TMD2, and conserved cysteines are shown as white letters on a black background. Disulfide bonds predicted by the crystal structure of cASIC1 are indicated by roman numbers (I-VII). Disulfide bond V is not conserved in closely related NeNaC1 and NeNaC24, and disulfide bond VI is not conserved in closely related NeNaC6 and NeNaC9. NeNaC10 lacks a stretch of about 25 amino acids in the middle of the ECD, including two conserved cysteines. This deletion is due to an alternative exon and likely renders this NeNaC non-functional. Putative positions of TMDs are depicted by black bars. The star indicates the DEG position, close to TMD2.

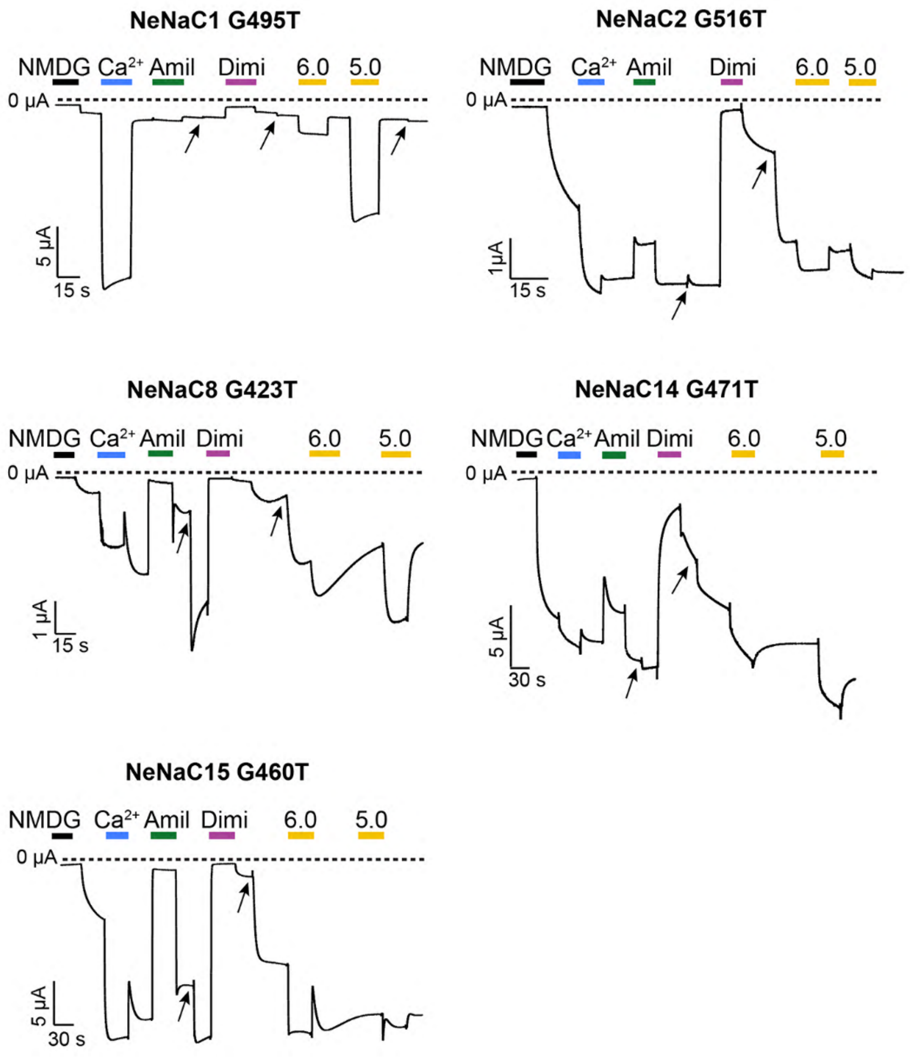


**Supplementary Figure 10. The DEG mutation constitutively activates some NeNaCs.** Depicted are representative traces of each mutant. They were all activated to a variable extent with the same stimuli: 10 µM $Ca^{2+}$ (blue), pH 6.0 and pH 5.0 (yellow). Moreover, they were blocked by 100 µM amiloride (green) or 10 µM diminazene (dark pink). Our automated solution exchange system exchanges approximately 99% of the bath solution. In some cases, for complete wash-out of blockers, we changed the bath solution twice (indicated by arrows).

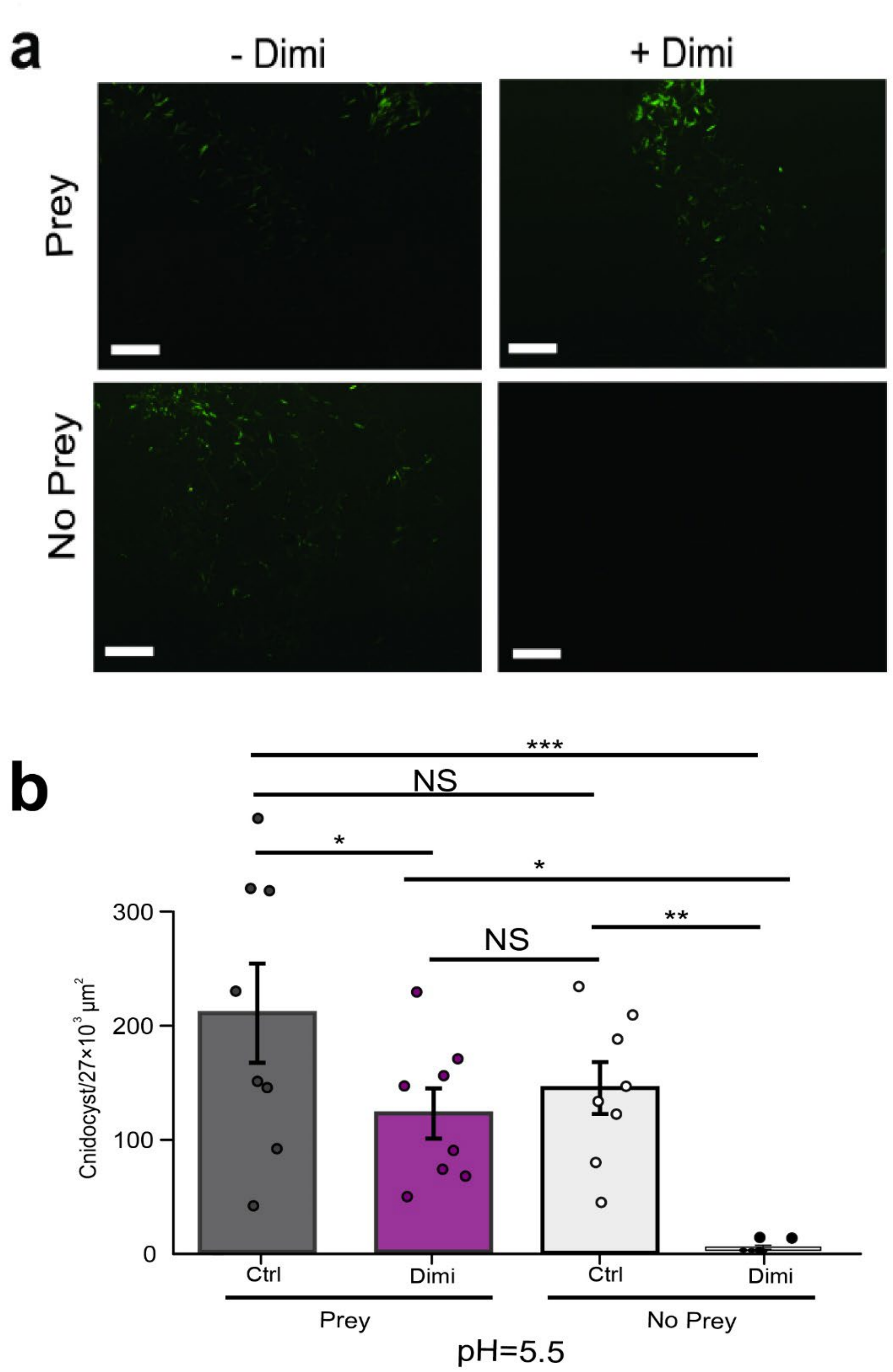


**Supplementary Figure 11. Cnidocyst discharge at pH 5.5. a** Pictures of discharged cnidocysts in *NvNCol3::mOrange2* positive organisms, with and without prey extract and with and without diminazene. Scale bar: 100 µm. **b** Bar graph showing the number of discharged cnidocysts (mean ± S.E.) at pH 5.5, with and without prey extract and with and without diminazene. One-way ANOVA from 8 individuals per treatment with Tukey post-hoc multiple comparisons test.

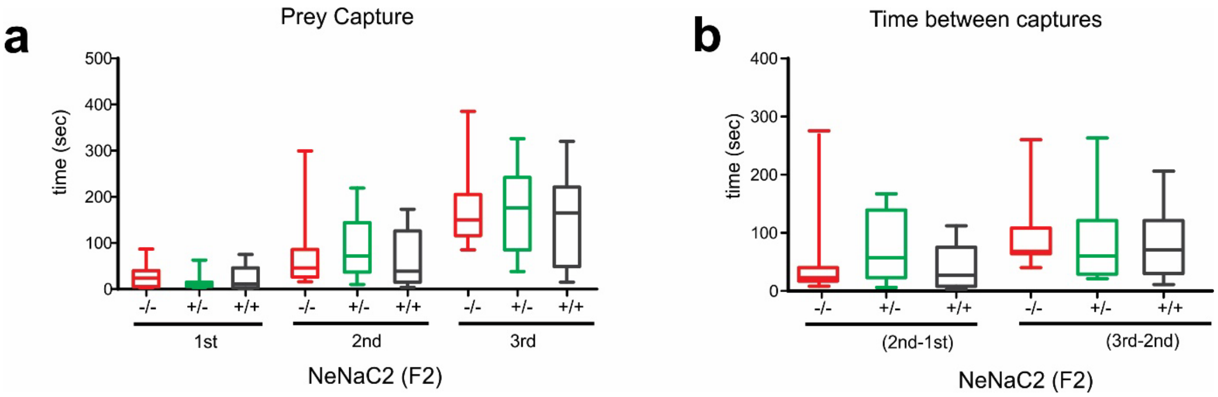


**Supplementary Figure 12. Prey capture experiment of NeNaC2 F2 progeny from the three genetic pools (NeNaC2$^{(+/+)}$, NeNaC2$^{(+/-)}$ and NeNaC2$^{(-/-)}$). a** Box plot with whiskers showing the time in seconds in which NeNaC2 F2 individuals from the three genetic pools capture the first, second and third nauplius at pH 7.2. **b** Box plot with whiskers showing the differences in time between the capture of the second and the first, and the third and the second nauplius at pH 7.2. One-way ANOVA from 15 individuals per treatment with Tukey post-hoc multiple comparisons test for data of **(a)** and **(b)**, and no significant differences were detected.

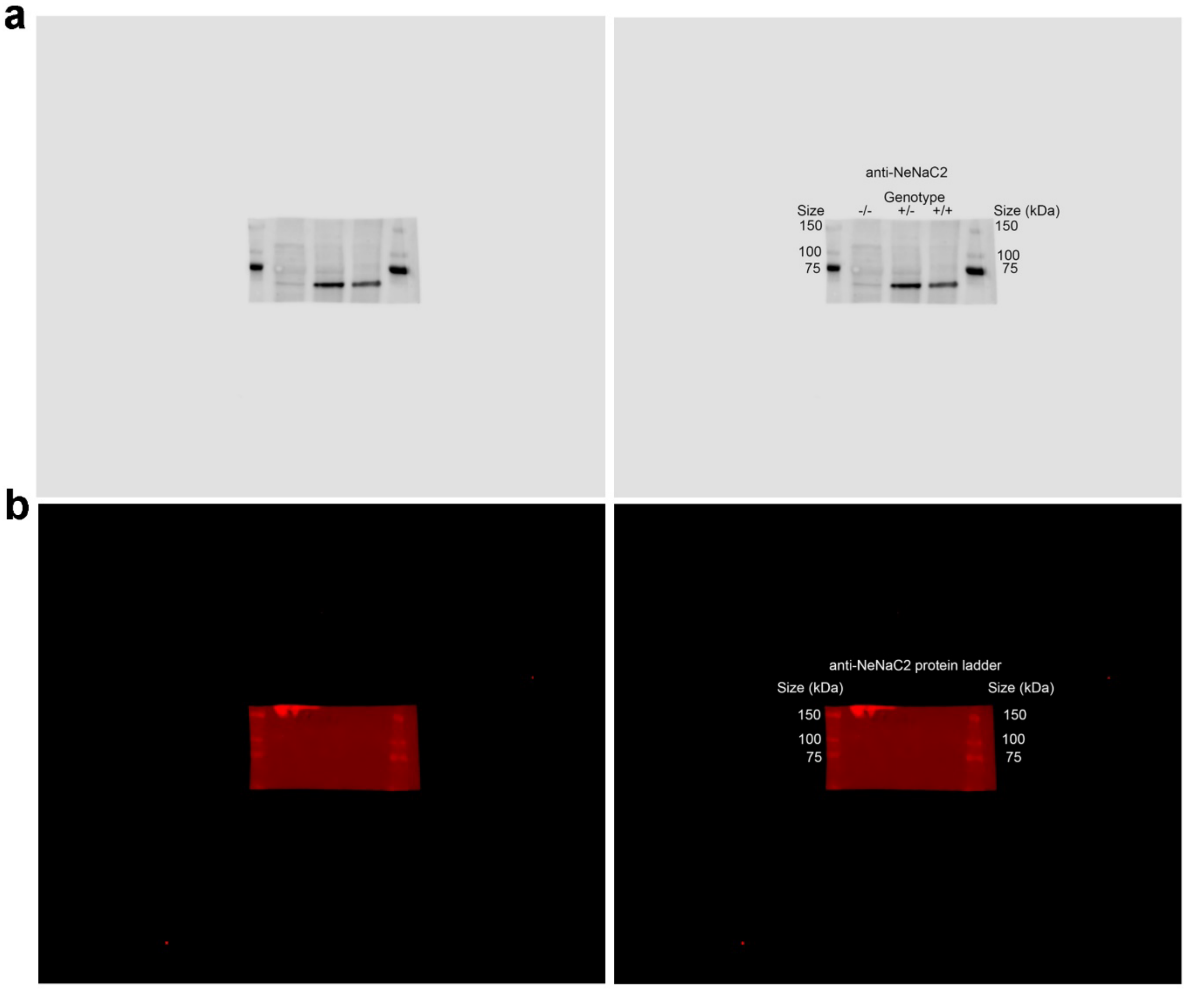


**Supplementary Figure 13. Unlabeled and labeled uncropped blot scans for anti-NeNaC2.** **a** Scan with CCD camera of the Odyssey Fc imaging system (Li-COR Biosciences, USA) of a western blot membrane incubated with anti-NeNaC2. **b** Scan of the same membrane with the infra-red detector of the same device for detecting the size marker. These scans were used for generating Figure 5i in the main text.

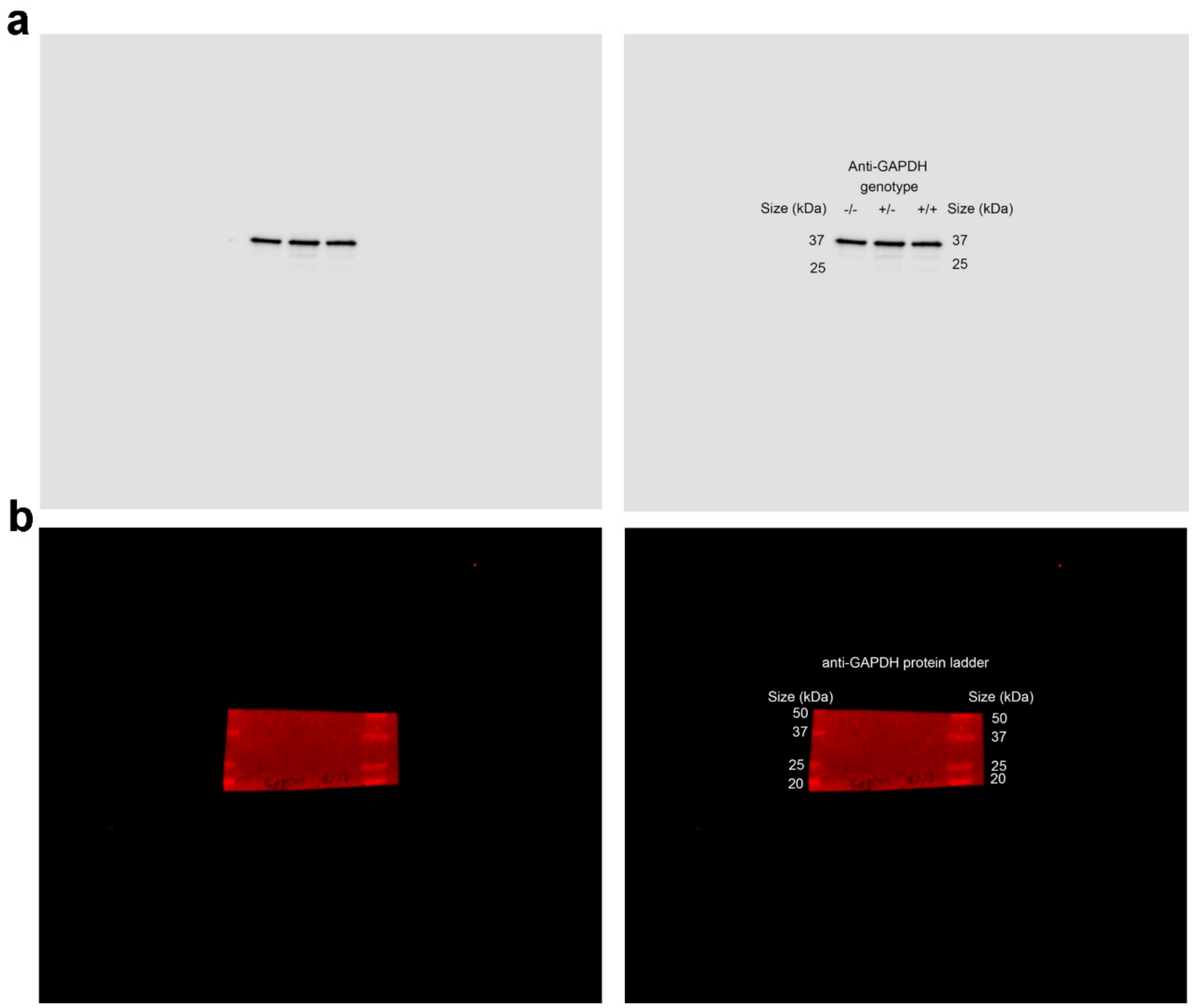


**Supplementary Figure 14. Unlabeled and labeled uncropped blot scans for anti-GAPDH.** **a** Scan with CCD camera of the Odyssey Fc imaging system (Li-COR Biosciences, USA) of a western blot membrane incubated with anti-GAPDH used as loading control. **b** Scan of the same membrane with the infra-red detector of the same device for detecting the size marker. These scans were used for generating Figure 5i in the main text.

## SUPPLEMENTARY REFERENCES


1. Anctil, M. Chemical transmission in the sea anemone *Nematostella vectensis*: A genomic perspective. *Comp. Biochem. Physiol. Part D Genomics Proteomics*. **4**, 268-289 (2009).
2. Koch, T. L. & Grimmelikhuijzen, C. J. P. A comparative genomics study of neuropeptide genes in the cnidarian subclasses Hexacorallia and Ceriantharia. *BMC Genom*. **21**, 666 (2020).
3. Hayakawa, E. et al. A combined strategy of neuropeptide prediction and tandem mass spectrometry identifies evolutionarily conserved ancient neuropeptides in the sea anemone *Nematostella vectensis*. *PLoS One.* **14**, e0215185 (2019).
