# Supplementary material for: Functional analysis in a model sea anemone reveals phylogenetic complexity and a role in cnidocyte discharge of DEG/ENaC ion channels
Source: Commun Biol. 2023 Jan 6;6:17. doi: 10.1038/s42003-022-04399-1 (PMC9822975; doi:10.1038/s42003-022-04399-1)
Supplement: Supplementary file 3 — Description of Additional Supplementary Files [file 42003_2022_4399_MOESM3_ESM.pdf]

Description of Additional Supplementary Files

File name: Supplementary Data 1

Description: The NeNaC amino acid sequences which were used for constructing phylogenetic trees in the paper

File name: Supplementary Data 2

Description: The NeNaC amino acid multiple sequence alignment which was used for constructing phylogenetic trees in the paper

File name: Supplementary Data 3

Description: The source data behind the graphs in the paper
